# A rotation and translation invariant method for 3D organ image classification using deep convolutional neural networks

**DOI:** 10.7717/peerj-cs.181

**Published:** 2019-03-04

**Authors:** Kh Tohidul Islam, Sudanthi Wijewickrema, Stephen O’Leary

**Affiliations:** Department of Surgery (Otolaryngology), University of Melbourne, Melbourne, Victoria, Australia

**Keywords:** Deep Learning, Medical Image Processing, Image Classification, Symmetry, 3D Organ Image Classification

## Abstract

Three-dimensional (3D) medical image classification is useful in applications such as disease diagnosis and content-based medical image retrieval. It is a challenging task due to several reasons. First, image intensity values are vastly different depending on the image modality. Second, intensity values within the same image modality may vary depending on the imaging machine and artifacts may also be introduced in the imaging process. Third, processing 3D data requires high computational power. In recent years, significant research has been conducted in the field of 3D medical image classification. However, most of these make assumptions about patient orientation and imaging direction to simplify the problem and/or work with the full 3D images. As such, they perform poorly when these assumptions are not met. In this paper, we propose a method of classification for 3D organ images that is rotation and translation invariant. To this end, we extract a representative two-dimensional (2D) slice along the plane of best symmetry from the 3D image. We then use this slice to represent the 3D image and use a 20-layer deep convolutional neural network (DCNN) to perform the classification task. We show experimentally, using multi-modal data, that our method is comparable to existing methods when the assumptions of patient orientation and viewing direction are met. Notably, it shows similarly high accuracy even when these assumptions are violated, where other methods fail. We also explore how this method can be used with other DCNN models as well as conventional classification approaches.

## Introduction

With the rapid growth of medical imaging technologies, a large volume of 3D medical images of different modalities such as magnetic resonance imaging (MRI), computed tomography (CT), and positron emission tomography (PET) has become available (Research & Markets, 2018). This has resulted in the formation of large medical image databases that offer opportunities for evidence-based diagnosis, teaching, and research. Within this context, the need for the development of 3D image classification methods has risen. For example, 3D medical image classification is used in applications such as computer aided diagnosis (CAD) and content-based medical image retrieval (CBMIR) (Zhou et al., 2006; Kumar et al., 2013).

In recent years, many algorithms have been introduced for the classification of 3D medical images (Arias et al., 2016; Mohan & Subashini, 2018). Both conventional classification methods and deep learning have been used for this purpose. For example, Öziç & Özşen (2017) proposed a voxel-based morphometry method to transform 3D voxel values into a vector to be used as features in a support vector machine (SVM) in order to identify patients with Alzheimer’s disease using MR images.

Bicacro, Silveira & Marques (2012) investigated the classification of 3D brain PET images into three classes: Alzheimer’s disease, mild cognitive impairment, and cognitively normal. To this end, they used three different feature extraction approaches (volumetric intensity, 3D Haar-like (Cui et al., 2007), and histogram of oriented gradients (HoG) (Dalal & Triggs, 2005)) and trained a SVM using these features.

Morgado, Silveira & Marques (2013) also performed a similar classification (Alzheimer’s disease, mild cognitive impairment, and cognitively normal) for PET brain images. They used 2D and 3D local binary patterns as texture descriptors to extract features and performed the classification task using a SVM.

A 3D image classification method was proposed by Liu & Dellaert (1998) for the pathological classification of brain CT images (captured by the same scanner) as normal, (evidence of) blood, or stroke. First, in a pre-processing step, they manually realigned all images so that the mid-sagittal plane was at the middle of the image. Then, considering the symmetry of the image, they extracted 50 image features from half of each 2D slice (in the superior-inferior direction) and used kernel regression for classification.

A limitation of conventional classification methods such as these, is that the most appropriate features for a given problem have to be extracted first, in order to train the classifiers. In contrast, deep learning techniques such as deep convolutional neural networks (DCNNs) extract the features as part of the learning process, thereby ensuring that the optimal features for a given task are extracted.

A 3D DCNN was used by Ahn (2017) to classify lung cancer (cancer positive or negative) from CT images. The author modified the SqueezeNet (Iandola et al., 2016) architecture (which is traditionally suitable for 2D images) to obtain SqueezeNet3D which is appropriate for 3D image classification.

Liu & Kang (2017) introduced a lung nodule classification approach by using a multi-view DCNN for CT images. They obtained a 3D volume by considering multiple views of a given nodule (patches of different sizes around the nodule) prior to classification. They performed two classifications: binary (benign or malignant) and ternary (benign, primary malignant, or metastatic malignant).

Jin et al. (2017) modified the AlexNet (Krizhevsky, Sutskever & Hinton, 2012) architecture to make it suitable for the classification of 3D CT images of lungs. They segmented the lungs from the CT image using a pre-processing step and performed a binary classification (cancer or not) on the resulting image. Instead of using 3D DCNNs, other researchers have considered how 2D DCNNs can be used to classify 3D medical images. For example, Qayyum et al. (2017) used each and every 2D slice of a 3D image as input to a 2D DCNN. They classified the images into 24 classes and used a publicly available 3D medical image database to evaluate their methodology.

Usually 3D medical images are captured/reconstructed so that they are consistent with respect to viewing direction and patient orientation (rotation and translation). For example, image slices are typically taken in the superior-inferior direction. An effort is made to ensure that the patient is aligned in such a way that the mid-sagittal and mid-coronal planes are aligned at the middle of the image. Thus, most classification methods assume that these requirements are met (e.g., Qayyum et al., 2017). As such, they may not perform well if these assumptions are violated. Others perform manual pre-processing prior to the classification to avoid this issue (e.g., Liu & Dellaert, 1998).

In this paper, we consider the specific case of 3D organ image classification and propose an algorithm that is robust against rotation and translation. To this end, we exploit the fact that the human body is roughly symmetric, and extract a 2D slice from the plane of best symmetry from a 3D image of the organ in a pre-processing step. We consider this slice to be representative of the 3D image, as it provides a relatively consistent cross-section of the 3D image, irrespective of its orientation. Then, we use this ‘representative’ 2D image to train a 2D DCNN to classify the 3D image. As discussed later, simplicity is one of the major features of the algorithm we propose.

We show through experiments performed on publicly available muliti-modal (CT and MRI) data that (1) the proposed method is as accurate as other similar methods when the above assumptions are met, (2) it significantly outperforms other methods when faced with rotated and/or translated data, (3) the training time of the proposed method is low, and (4) it achieves similarly high results when used with other DCNN architectures.

## Materials and Methods

In this section, we discuss the steps of the algorithm we propose for rotation and translation invariant 3D organ image classification: volume reconstruction, segmentation, symmetry plane extraction, and classification using a DCNN.

### Volume reconstruction

**Figure 1:**
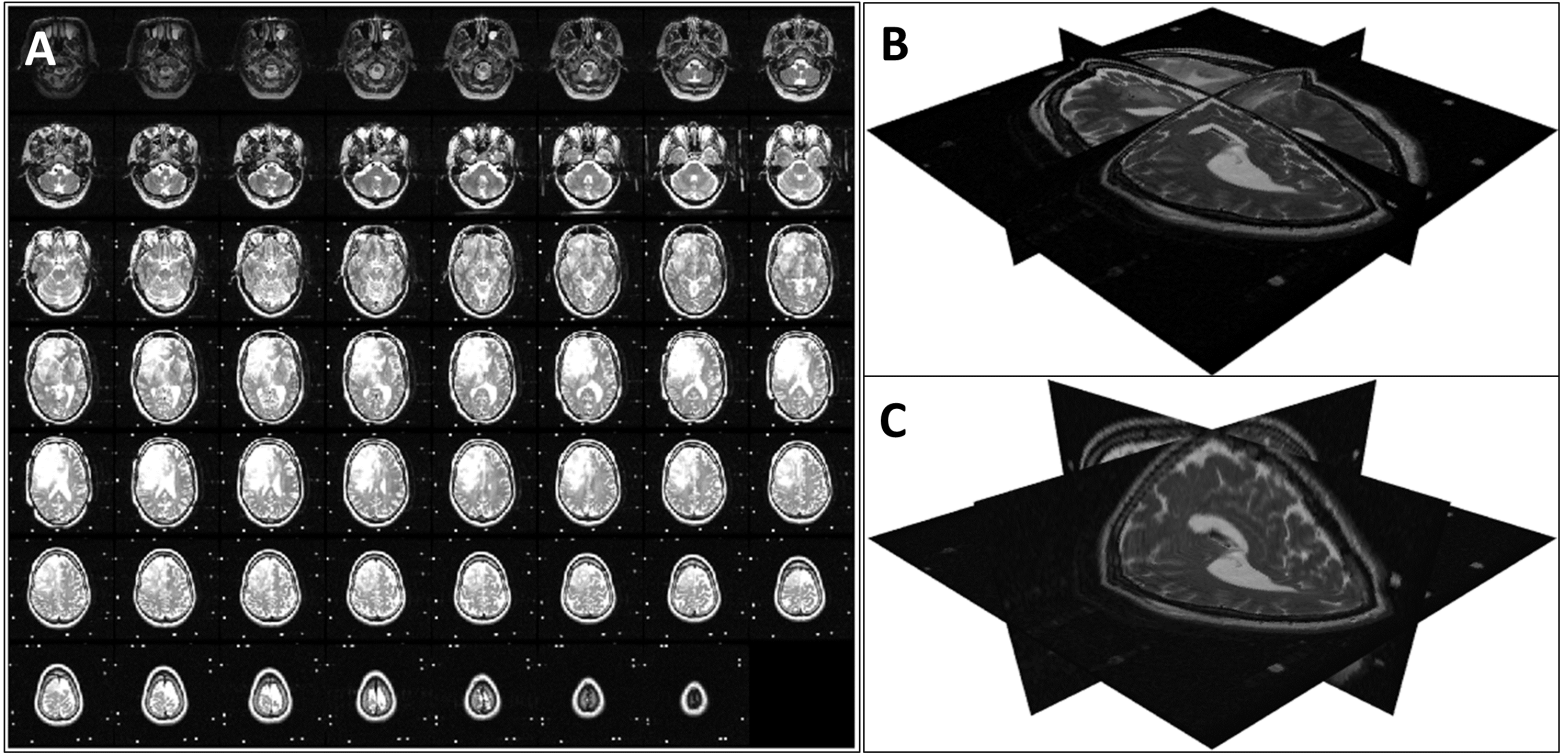
Volume reconstruction from DICOM images: (A) stack of 2D slices, (B) reconstructed 3D volume, and (C) resampled 3D volume.

First, we loaded the 2D slices of a DICOM image into a 3D array considering the InstanceNumber in the metadata to be the *z* dimension. As the slice thickness (*z* spacing) is not necessarily the same as the pixel spacing, this volume does not represent the real-world shape of the imaged organ/body part. To retain the actual shape, we resampled the 3D image using cubic interpolation (Miklos, 2004). The new array size for the resampled image was calculated using Eq. (1) where [*n*_*x*_, *n*_*y*_, *n*_*z*_] is the original array size, *ps*_*x*_ and *ps*_*y*_ are the *x* and *y* spacings respectively (PixelSpacing in metadata), and *st* is the *z* spacing (SliceThickness in metadata). An example of a volume reconstruction is shown in Fig. 1.

(1) \begin{eqnarray*}[{n}_{x},{n}_{y},{n}_{z}]& =& \left[ \right. {n}_{x}, \frac{{n}_{y}\ast p{s}_{y}}{p{s}_{x}} , \frac{{n}_{z}\ast st}{p{s}_{x}} \left( \right. \end{eqnarray*}

### 3D volume segmentation

**Figure 2:**
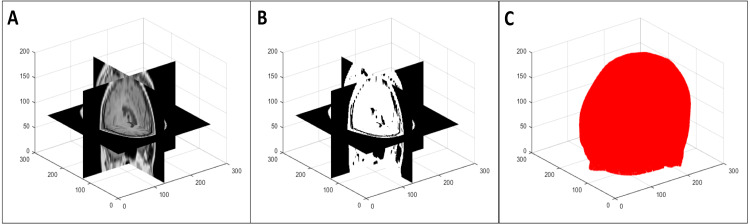
Multi-level volume thresholding: (A) resampled volume from the previous step, (B) segmented volume, and (C) resulting point cloud.

To segment the organ(s) from the background, we used a multi-level global thresholding using Otsu’s method (Otsu, 1979). We used two thresholds and considered the voxels with intensity values within these thresholds to be the organ(s). This provides a segmentation (point cloud) of the organ(s) and also avoids the inclusion of possible imaging artifacts at the extremes of the intensity spectrum. An example of the segmentation process is shown in Fig. 2. Note that this is a simple segmentation process, and as such, does not provide an exact segmentation. However, from our results, we observed that this was sufficient for our purpose: simplifying the symmetry plane calculation in the next step of our algorithm.

### Representative 2D image extraction

We calculated the plane of best symmetry from the point cloud resulting from the previous step using the method discussed in Cicconet, Hildebrand & Elliott (2017). They calculated the reflection of a point cloud around an arbitrary plane, used the iterative closest point algorithm (Besl & McKay, 1992) to register the original and reflected point clouds, and solved an eigenvalue problem related to the global transformation that was applied to the original data during registration. The first eigenvector in this solution is the normal to the plane of best symmetry.

**Figure 3:**
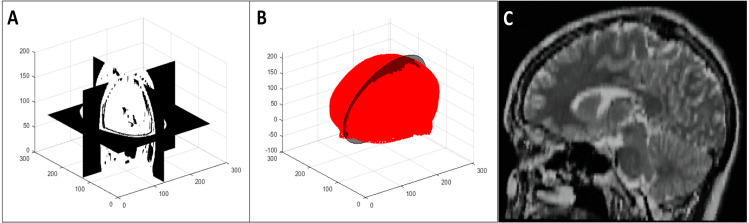
Representative 2D image extraction: (A) segmented volume, (B) segmented point cloud with the plane of best symmetry shown as a circular plane, and (C) 2D image extracted from the symmetry plane.

We extracted the 2D image resulting from the intersection of this plane with the 3D volume using the nearest neighbour method (Miklos, 2004). We considered the second and third eigenvectors to be the *x* and *y* axes for this 2D image respectively. We determined the bounds of the 2D image to be the minimum and maximum values resulting from the projections of the 3D volume vertices on the axes and the origin to be the middle of these minimum and maximum values. Figure 3 shows the extraction of the plane of best symmetry.

Although the accuracy of the symmetry plane calculation depends on the segmentation step, and this can be avoided by using algorithms that minimize the distance between intensity values of voxels (Tuzikov, Colliot & Bloch, 2003; Teverovskiy & Li, 2006) instead, using the segmented point cloud is more efficient. As we found it is sufficient for our purposes, we used this method of symmetry plane calculation for the sake of efficiency.

### Classification using a DCNN

**Figure 4:**
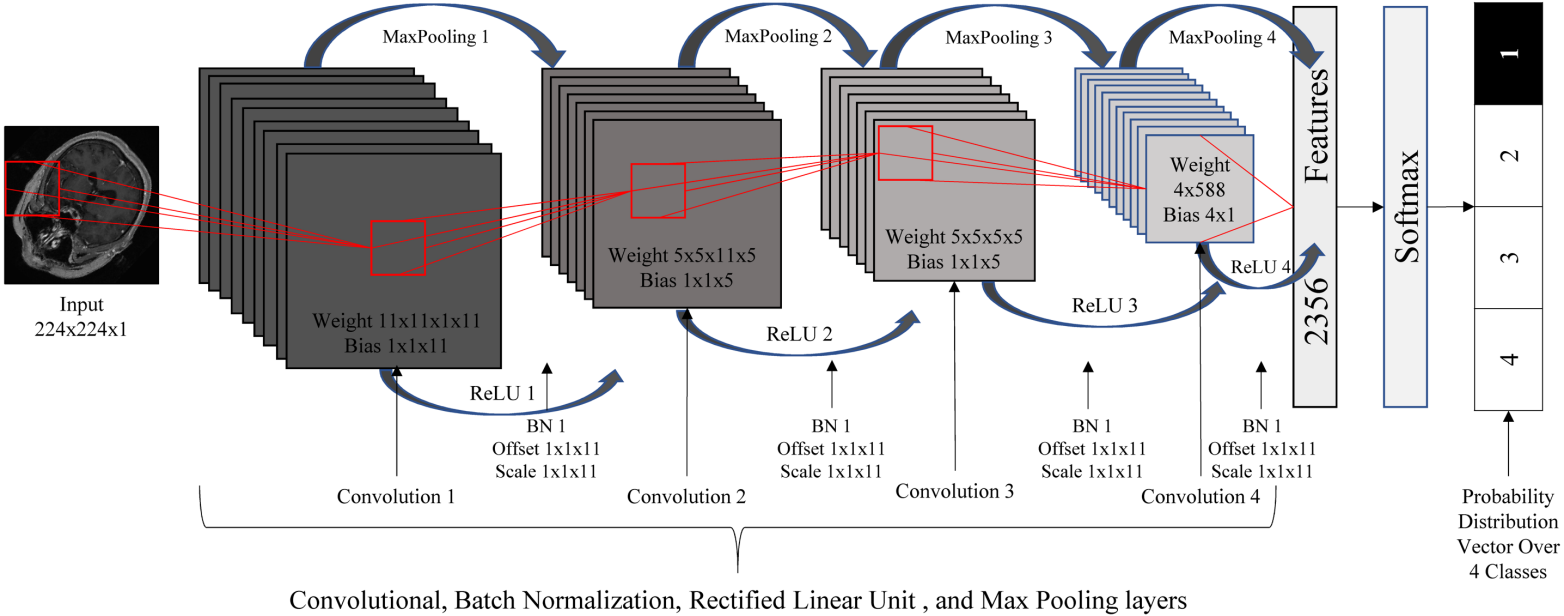
Deep convolutional neural network architecture.

Due to the roughly symmetric nature of the human body, the 2D images resulting from the previous step provide relatively consistent cross-sections of the 3D images. As such, we used these 2D images to train a standard 2D DCNN for the classification task. The DCNN used here consisted of 20 layers: one image input layer, four convolution layers, four batch normalization layers, four rectified linear unit (ReLU) (Nair & Hinton, 2010) layers, four max poling layers, one fully connected layer, one softmax layer, and one classification output layer. We resized the images to the size of 224 × 224 and normalized the intensity values to be in the range of [0 255]. Figure 4 illustrates the DCNN architecture.

## Results

In this section, we discuss the performance metrics and databases used in the experiments, the implementation/extension of existing methods, and the experimental results. All experiments were performed in MATLAB^^®^^ (MathWorks Inc., 1998) on a HP Z6 G4 Workstation running Windows^^®^^10 Education on an Intel^^®^^Xeon^^®^^Silver 4108 CPU with a clock speed of 1.80 GHz, 16 GB RAM, and a NVIDIA^^®^^Quadro^^®^^P2000 GPU.

### Performance evaluation metrics

To evaluate classification performance, we used commonly utilized metrics (accuracy and mean value of sensitivity, specificity, precision, f-measure, and g-mean) (Japkowicz, 2006; Powers, 2011; Olson & Delen, 2008). These metrics are defined in Eqs. (2)–(7) with respect to values of the confusion matrix: true positives (*TP*), true negatives (*TN*), false positives (*FP*), and false negatives (*FN*).

(2) \begin{eqnarray*}Accuracy& =& \frac{TP+TN}{TP+FN+FP+TN} \end{eqnarray*}

(3) \begin{eqnarray*}Sensitivity& =& \frac{TP}{TP+FN} \end{eqnarray*}

(4) \begin{eqnarray*}Specificity& =& \frac{TN}{TN+FP} \end{eqnarray*}

(5) \begin{eqnarray*}Precision& =& \frac{TP}{TP+FP} \end{eqnarray*}

(6) \begin{eqnarray*}F-Measure& =& 2\times \left( \right. \frac{ \frac{TP}{TP+FP} \times \frac{TP}{TP+FN} }{ \frac{TP}{TP+FP} + \frac{TP}{TP+FN} } \left( \right. \end{eqnarray*}

(7) \begin{eqnarray*}G-Mean& =& \sqrt{ \frac{TP}{TP+FN} \times \frac{TN}{TN+FP} }\end{eqnarray*}

### Databases

We collected data from a publicly available 3D medical image database for our experiments: the cancer imaging archive (TCIA) (Clark et al., 2013). TCIA stores a large number of multi-modal medical images stored in the digital imaging and communications in medicine (DICOM) file format. From this database, we collected data (CT and MRI) for four classes that define different areas of the human body: head, thorax, breast, and abdomen. Some images, such as those that had a very low number of 2D DICOM slices and those with inconsistent imaging directions, were removed from our database. A total of 2400 3D images were obtained (600 images per class).

**Figure 5:**
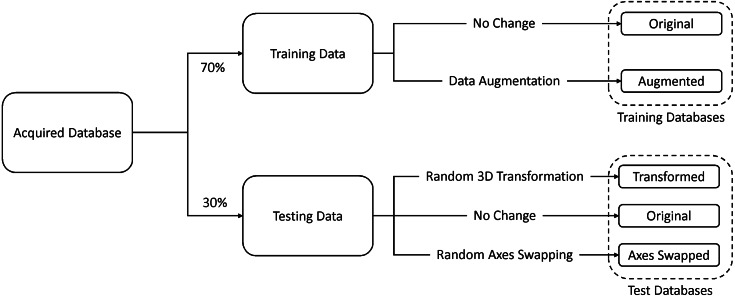
Database formation.

Seventy percent of the images were used for training and the remaining thirty percent were used for testing. In addition to the original testing database, we created two other databases by (1) randomly rotating and translating and (2) randomly swapping the axes of the original test data. The former database was used to test for rotation and translation (patient orientation) invariance and the latter was used to test for robustness against changes in the imaging direction. In addition to this, we created an augmented training database by randomly rotating and translating 50% of the original training data, and randomly swapping axes of the remaining 50%. Figure 5 illustrates this process.

**Figure 6:**
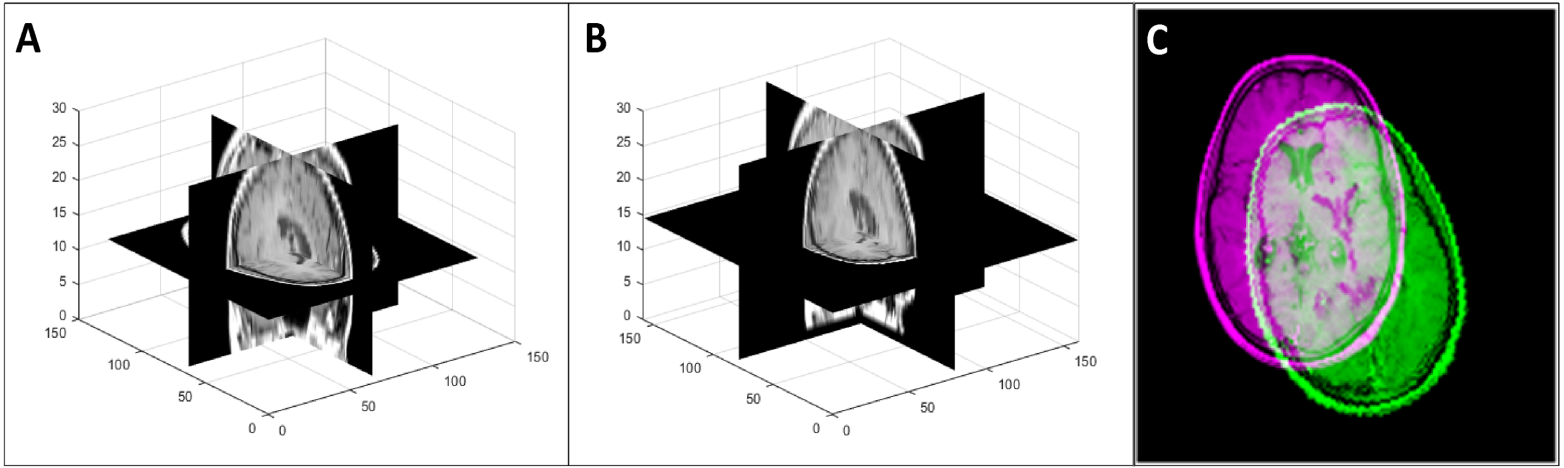
An example of a random 3D transformation: (A) original volume, (B) transformed volume (a rotation of +15 ^0^ counterclockwise around the *z* axis and a translation of [3, 2, 2] in the *x*, *y*, and *z* direction respectively), and (C) mid-axial slices of both original and transformed volumes.

To generate the transformed data that simulated changes in patient orientation (in the transformed test database and the augmented training database), we performed a random rotation in the range of [−15^0^ 15^0^] with respect to the three coordinate axes and a random translation of [−5 5] along the coordinate axes on each image. Figure 6 shows an example of such a 3D transformation.

**Figure 7:**
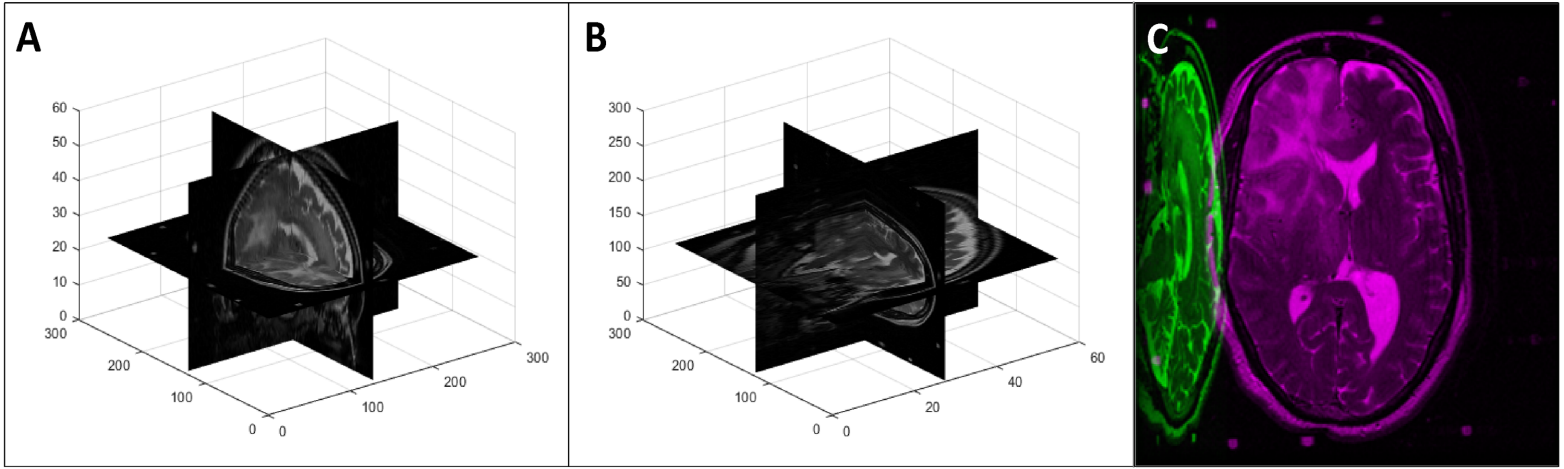
An example of a random 3D axis swapping: (A) original volume, (B) axis swapped volume with the *x* axis changed to the *y* axis and *z* axis changed to the *x* axis, and (C) mid-axial slices of both original and axis swapped volumes.

To obtain the axis swapped data that simulated changes in imaging direction (in the axis swapped test database and the augmented training database), we randomly changed the axes of the original data. Note that this is synonymous to rotations of 90^0^ around the *x*, *y*, or *z* axis. An example of a random axis swapping is shown in Fig. 7.

### Performance comparison with other similar methods

We evaluated our method against similar existing methods. We reimplemented the method of Qayyum et al. (2017) that used all 2D slices to represent the 3D volume. We implemented their DCNN in MATLAB and used all slices of the training and testing sets respectively to train and evaluate this method. As the authors used images of size 224 × 224 as their input, we also performed the same resizing of the data in a pre-processing step.

We also implemented the method used in the classification of 3D lung images introduced in Jin et al. (2017). They used thresholds based on the Hounsfield unit (HU) to extract an initial mask from CT images and used morphological operations to fill holes in this mask. Then, they segmented the lungs using this mask and trained a DCNN for the classification task. However, this method cannot be directly used in our problem. First, we have multi-modal data, and hence, it is not possible to use the HU scale, which is specific to CT images. Second, we have images of different organs which would require the definition of organ specific thresholds. Third, morphological operations require the input of the size of dilation/erosion which varies depending on the type of image.

Therefore, we used a process that can be generally applied to all images in our database. First, we created a binary mask using the multi-level global thresholding method discussed earlier. Then, we used active contours introduced in Chan & Vese (2001) on the initial mask with 100 iterations to fill in holes and obtain a more refined mask. Finally, we extracted the organ from the background using this mask and used this as the input to the DCNN. Jin et al. (2017) observed that an input image size of 128 × 128 × 20 provided the best performance, and therefore, we also resized the input images to this size.

**Table 1:** Performance comparison with similar existing methods (without data augmentation). Best performance per metric per database is highlighted in bold.

**Database**	**Methodology**	**Accuracy**	**Sensitivity**	**Specificity**	**Precision**	**F-Measure**	**G-Mean**
Original	Jin et al. (2017)	0.9514	0.9514	0.9838	0.9525	0.9515	0.9675
	Qayyum et al. (2017)	0.9014	0.9014	0.9671	0.9168	0.9029	0.9337
	Prasoon et al. (2013)	0.9653	0.9653	0.9884	0.9679	0.9654	0.9768
	Ahn (2017)	**0.9972**	**0.9972**	0.9991	**0.9973**	**0.9972**	**0.9981**
	Proposed	0.9944	0.9944	0.9981	0.9945	0.9944	0.9963
Transformed	Jin et al. (2017)	0.8611	0.8611	0.9537	0.9080	0.8642	0.9062
	Qayyum et al. (2017)	0.6694	0.6694	0.8898	0.7670	0.5921	0.7718
	Prasoon et al. (2013)	0.9306	0.9306	0.9769	0.9364	0.9306	0.9534
	Ahn (2017)	0.9333	0.9333	0.9778	0.9378	0.9325	0.9553
	Proposed	**0.9917**	**0.9917**	**0.9972**	**0.9919**	**0.9916**	**0.9944**
Axis Swapped	Jin et al. (2017)	0.6222	0.6222	0.8741	0.7549	0.6065	0.7375
	Qayyum et al. (2017)	0.5056	0.5056	0.8352	0.7630	0.4491	0.6498
	Prasoon et al. (2013)	0.8028	0.8028	0.9343	0.8420	0.7936	0.8660
	Ahn (2017)	0.7264	0.7264	0.9088	0.7707	0.7122	0.8125
	Proposed	**0.9875**	**0.9875**	**0.9958**	**0.9876**	**0.9875**	**0.9917**

**Table 2:** Performance comparison with similar existing methods (with data augmentation by random transformation and axis swapping on training data). Best performance per metric per database is highlighted in bold.

**Database**	**Methodology**	**Accuracy**	**Sensitivity**	**Specificity**	**Precision**	**F-Measure**	**G-Mean**
Original	Jin et al. (2017)	0.9222	0.9222	0.9741	0.9225	0.9213	0.9478
	Qayyum et al. (2017)	0.9042	0.9042	0.9681	0.9082	0.9037	0.9356
	Prasoon et al. (2013)	0.9375	0.9375	0.9792	0.9387	0.9370	0.9581
	Ahn (2017)	0.9597	0.9597	0.9866	0.9607	0.9595	0.9731
	Proposed	**0.9903**	**0.9903**	**0.9968**	**0.9903**	**0.9903**	**0.9935**
Transformed	Jin et al. (2017)	0.9139	0.9139	0.9713	0.9139	0.9132	0.9422
	Qayyum et al. (2017)	0.8931	0.8931	0.9644	0.9023	0.8917	0.9280
	Prasoon et al. (2013)	0.9306	0.9306	0.9769	0.9314	0.9296	0.9534
	Ahn (2017)	0.9375	0.9375	0.9792	0.9409	0.9368	0.9581
	Proposed	**0.9681**	**0.9681**	**0.9894**	**0.9682**	**0.9678**	**0.9786**
Axis Swapped	Jin et al. (2017)	0.8903	0.8903	0.9634	0.8910	0.8894	0.9261
	Qayyum et al. (2017)	0.8597	0.8597	0.9532	0.8811	0.8604	0.9053
	Prasoon et al. (2013)	0.9028	0.9028	0.9676	0.9039	0.9018	0.9346
	Ahn (2017)	0.9194	0.9194	0.9731	0.9222	0.9190	0.9459
	Proposed	**0.9653**	**0.9653**	**0.9884**	**0.9658**	**0.9652**	**0.9768**

Another 3D medical image classification model we implemented was Ahn (2017) which was used for lung image classification. The author performed an intensity normalization of their CT images based on the HU scale in a pre-processing step. Due to the same reasons as above, we did not perform this normalization. We used the same resizing of the data they used (128 × 128 × 128) in our implementation.

As an additional method of comparison, we extended the idea presented in Prasoon et al. (2013) for image segmentation, to make it applicable to our problem. They explored the classification of each voxel in 3D MRI images for the purpose of knee cartilage segmentation. They extracted three 2D patches around a voxel in the *x*, *y*, and *z* directions, trained DCNNs for each 2D patch, and combined the results of the three DCNNs in the final layer. We applied this idea to our problem by extracting the mid slices in the three coordinate directions and training three DCNNs similar to theirs.

**Figure 8:**
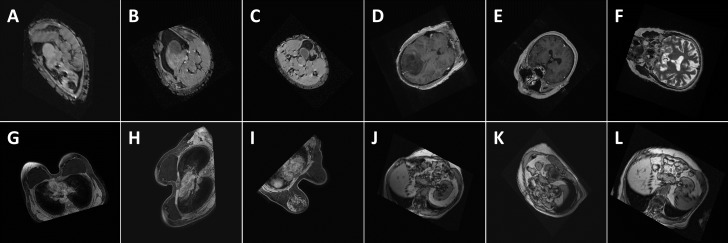
Performance (confidence level of classification) of the proposed method with respect to some random images: (A) Abdomen, 85.7%, (B) Abdomen, 92.3%, (C) Abdomen, 94.2%, (D) Head, 99.9%, (E) Head, 100%, (F) Head, 98.8%, (G) Breast, 100%, (H) Breast, 100%, (I) Breast, 100%, (J) Thorax, 98.9%, (K) Thorax, 98.9%, and (L) Thorax, 99.7%. The images show the views extracted using the proposed algorithm from 3D CT and MRI images in the test databases. Note that the differences in the images of the same class are caused by the simplicity of the segmentation method which influences the symmetry plane extraction.

Performance comparisons with respect to the metrics discussed above when trained on the original and augmented training datasets are shown in Tables 1 and 2 respectively. Performance with regards to training time is given in Table 3. Figure 8 shows the classification of some random examples using the proposed method, along with corresponding confidence levels.

**Table 3:** Comparison of training time with similar existing methods.

**Method**	**Pre-processing Time (m)**	**Training Time (m)**	**Total Time (m)**
Jin et al. (2017)	240	1230	1470
Qayyum et al. (2017)	N/A	2732	2732
Prasoon et al. (2013)	1012	23	1035
Ahn (2017)	27	7252	7279
Proposed	232	20	252

### Performance when used with other DCNNs

We also investigated the performance of our method when used with some existing state-of-the-art DCNNs: AlexNet (Krizhevsky, Sutskever & Hinton, 2012), GoogLeNet (Szegedy et al., 2015), ResNet-50 (He et al., 2016), and VGG-16 (Simonyan & Zisserman, 2014). To enable these DCNNs to be used in our algorithm, we normalized the 2D images (extracted from the plane of best symmetry) prior to the classification depending on the requirements of each DCNN. The single channel 2D grey scale images were converted to three-channel colour images and resized to the size of 127 × 127 × 3 for AlexNet and 224 × 224 × 3 for GoogLeNet, ResNet-50, and VGG-16. The performance results are shown in Table 4.

**Table 4:** Performance of the proposed algorithm when used with other state-of-the-art DCNN models.

**Database**	**Methodology**	**Accuracy**	**Sensitivity**	**Specificity**	**Precision**	**F-Measure**	**G-Mean**
Original	AlexNet	0.9958	0.9958	0.9986	0.9958	0.9958	0.9972
	GoogLeNet	0.9986	0.9986	0.9995	0.9986	0.9986	0.9991
	ResNet-50	0.9972	0.9972	0.9991	0.9973	0.9972	0.9981
	VGG-16	0.9708	0.9708	0.9903	0.9717	0.9708	0.9805
Transformed	AlexNet	0.9944	0.9944	0.9981	0.9945	0.9944	0.9963
	GoogLeNet	0.9931	0.9931	0.9977	0.9931	0.9931	0.9954
	ResNet-50	0.9958	0.9958	0.9986	0.9958	0.9958	0.9972
	VGG-16	0.9653	0.9653	0.9884	0.9664	0.9653	0.9768
Axis Swapped	AlexNet	0.9931	0.9931	0.9977	0.9931	0.9931	0.9954
	GoogLeNet	0.9917	0.9917	0.9972	0.9917	0.9916	0.9944
	ResNet-50	0.9931	0.9931	0.9977	0.9930	0.9930	0.9954
	VGG-16	0.9639	0.9639	0.9880	0.9650	0.9640	0.9759

### Performance when used with conventional classifiers

As conventional classification approaches, in concert with image feature extraction methods, have been used extensively for image classification, we also explored how to integrate the concepts discussed here with these methods. For this purpose, we used two image feature extraction methods: bag of words (BoW) (Harris, 1954) and histogram of oriented gradients (HoG) (Dalal & Triggs, 2005). To perform the classification task, we used support vector machines (SVMs) and artificial neural networks (ANNs) as they are widely used in classification. Here we used five-fold cross-validation for the SVM and 10 hidden neurons for the ANN. We normalised the 2D image slices resulting from the symmetry plane calculation to the size of 224 × 224. The performance of these approaches is shown in Table 5.

**Table 5:** Performance of the proposed algorithm when used with conventional machine learning approaches.

**Database**	**Methodology**	**Accuracy**	**Sensitivity**	**Specificity**	**Precision**	**F-Measure**	**G-Mean**
Original	BoW+SVM	0.8083	0.8083	0.9361	0.8112	0.8047	0.8699
	BoW+ANN	0.7667	0.7667	0.9222	0.7927	0.7568	0.8409
	HoG+SVM	0.8500	0.8500	0.9500	0.8648	0.8470	0.8986
	HoG+ANN	0.7472	0.7472	0.9157	0.7983	0.7221	0.8272
Transformed	BoW+SVM	0.7944	0.7944	0.9315	0.7992	0.7908	0.8602
	BoW+ANN	0.7542	0.7542	0.9181	0.7820	0.7423	0.8321
	HoG+SVM	0.8375	0.8375	0.9458	0.8549	0.8334	0.8900
	HoG+ANN	0.7458	0.7458	0.9153	0.8113	0.7240	0.8262
Axis Swapped	BoW+SVM	0.7903	0.7903	0.9301	0.7923	0.7864	0.8573
	BoW+ANN	0.7500	0.7500	0.9167	0.7773	0.7350	0.8292
	HoG+SVM	0.8361	0.8361	0.9454	0.8556	0.8324	0.8891
	HoG+ANN	0.7361	0.7361	0.9120	0.7982	0.7136	0.8194

## Discussion

From the results in Table 1, we observe that the proposed method is better than other similar methods (except for Ahn (2017) which shows slightly better performance) when applied to data that satisfied the conditions of consistent patient orientation and imaging direction when trained on the original (unaugmented) training database. However, Ahn (2017) uses 3D DCNNs in their classification and therefore, has a much slower training time when compared to the proposed method. As shown in Table 3, even with a relatively higher pre-processing time, our method is the fastest in terms of total training time.

Also from Table 1, we can see that the proposed method outperforms the other methods in the face of changes in patient orientation and imaging direction. Although observe that some methods such as Ahn (2017) and Prasoon et al. (2013) are robust against patient orientation to some degree, they also fail when dealing with changes to imaging direction.

Performance of the compared methods on transformed and axis swapped data is improved when trained on augmented data, as seen in Table 2. This is the result of the classifiers being trained on images of different orientations. However, the proposed method outperforms the other methods even when training was performed on augmented data. Also, the results imply that data augmentation in the training phase is not required for our method.

The high accuracy of the proposed method, specifically on transformed data, is mainly due to the fact that a relatively consistent 2D cross-sectional view of a 3D image is being used to represent the 3D image irrespective of orientation. As such, the variation in the input data per class is minimal and therefore, better classification can be achieved.

Comparison results shown in Table 3 reflect the robustness of the proposed method irrespective of the DCNN architecture used in the classification step. The performance results of the classifiers SVM and ANN, when combined with the feature extraction methods of BoW and HoG, show consistent but lower results (Table 4). This indicates that DCNNs may be better suited for our application.

The salient feature of this algorithm is its simplicity. First, we reduced the 3D classification problem to a 2D one by extracting the 2D image lying on the plane of best symmetry from the 3D volume. In this operation, we used calculations that were most efficient, such as simple thresholding techniques. It can be argued that using more sophisticated methods of segmentation would enable more accurate symmetry plane calculation, which in turn would make the 2D views extracted more consistent. Furthermore, we rescaled the data to an 8-bit representation (intensity range of [0 255]), thereby reducing the resolution of the data. However, we found that even in the face of such simplifications, the proposed method displayed very high levels of performance. As such, we can conclude that it has achieved a good balance between efficiency and accuracy.

Although the human body is roughly symmetric, most of the organs and how they are aligned inside the body are not perfectly symmetrical. Furthermore, the data we considered here was from a cancer database where there is further asymmetry caused by tumors, lesions etc. Our method was observed to perform well in these circumstances. However, we did not consider the effect of more exaggerated forms of asymmetry, for example, that caused by parts of an organ being cut off due to improper patient alignment. In the future, we will investigate how these forms of asymmetry affect the proposed method and how to compensate for them. We will also explore how it performs on other databases with higher numbers of classes.

## Conclusion

In this paper, we proposed a 3D organ image classification approach which is robust against patient orientation and changes in imaging direction. To this end, we extracted the plane of best symmetry from the 3D image and extracted the 2D image corresponding to that plane. Then, we used a DCNN to classify the 2D image into one of four classes. We showed that this method is not only efficient and simple, but is also highly accurate in comparison to other similar methods. We also showed that this algorithm can be used in concert with other state-of-the-art DCNN models and also conventional classification techniques in combination with feature extraction methods. Although our algorithm was specifically developed for 3D organ image classification, it is applicable to any classification task where a 2D image extracted from the plane of best symmetry of the 3D image is sufficient to represent the 3D image.

##  Supplemental Information

10.7717/peerj-cs.181/supp-1 Supplemental Information 1 Raw Codes

## Additional Information and Declarations

### Data Availability

The following information was supplied regarding data availability: The code is available in the Supplemental Information 1.
